# Subjectivity “Demystified”: Neurobiology, Evolution, and the Explanatory Gap

**DOI:** 10.3389/fpsyg.2019.01686

**Published:** 2019-07-31

**Authors:** Todd E. Feinberg, Jon Mallatt

**Affiliations:** ^1^Icahn School of Medicine at Mount Sinai, Psychiatry and Neurology, New York, NY, United States; ^2^The University of Washington WWAMI Medical Education Program at The University of Idaho, Moscow, ID, United States

**Keywords:** primary consciousness, phenomenal consciousness, explanatory gap, neurobiology, subjectivity, evolution

## Abstract

While life in general can be explained by the mechanisms of physics, chemistry, and biology, to many scientists and philosophers, it appears that when it comes to explaining consciousness, there is what the philosopher Joseph Levine called an “explanatory gap” between the physical brain and subjective experiences. Here, we deduce the living and neural features behind primary consciousness within a naturalistic biological framework, identify which animal taxa have these features (the vertebrates, arthropods, and cephalopod molluscs), then reconstruct when consciousness first evolved and consider its adaptive value. We theorize that consciousness is based on all the complex system features of life, plus even more complex features of elaborate brains. We argue that the main reason why the explanatory gap between the brain and experience has been so refractory to scientific explanation is that it arises from both life and from varied and diverse brains and brain regions, so bridging the gap requires a complex, multifactorial account that includes the great *diversity* of consciousness, its *personal nature* that stems from embodied life, and the special neural features that make consciousness *unique* in nature.

## Introduction

C.D. Broad in his classic book, *The Mind and its Place in Nature*, presented the difficulty of understanding how observable biological facts about the brain could explain experience. He described a thought experiment where he argued that even if an omniscient “mathematical archangel” had total knowledge of the chemistry of ammonia and the functions of the brain, the archangel still would not be able to explain or predict the *smell* of ammonia:

He [the archangel] would know exactly what the microscopic structure of ammonia must be; but he would be totally unable to predict that a substance with this structure must smell as ammonia does when it gets into the human nose. The utmost that he could predict on this subject would be that certain changes would take place in the mucous membrane, the olfactory nerves and so on. But he could not possibly know that these changes would be accompanied by the appearance of a smell in general or of the peculiar smell of ammonia in particular, unless someone told him so or he had smelled it for himself (Broad, 1925, p. 71; also see Jackson, 1986).

Levine (1983) called the apparent divide between objective explanations of brain functions and their accompanying subjective feelings the *explanatory gap.* In the following quote from his paper, he applies the gap to the lack of a clear connection between the objective neurons that transmit pain (C-fibers) and the subjective experience of pain itself:

However, there is more to our concept of pain than its causal role, there is its qualitative character, how it feels; and what is left unexplained by the discovery of C-fiber firing is *why pain should feel the way it does*! For there appears to be nothing about C-fiber firing which makes it naturally “fit” the phenomenal properties of pain, any more than it would fit some other set of phenomenal properties… The identification of the qualitative side of pain with C-fiber firing (or some property of C-fiber firing) leaves the connection between it and what we identify it with completely mysterious. One might say, it makes the way pain feels into merely brute fact (Levine, 1983, p. 357).

The subjective, qualitative aspect of consciousness that concerns Levine has been called *phenomenal consciousness, primary consciousness,* *sensory* *consciousness,* having any feelings, and experiencing *phenomenal properties* (which are perceived qualities, alternately called the *phenomenal characters of experience* and *qualia*). As defined by Antti Revonsuo, this basic form of sensory experience does not have to be elaborate, lingering, reflective, or human-like to be conscious:

The mere occurrence or presence of any experience is the necessary and minimally sufficient condition for phenomenal consciousness. For any entity to possess primary phenomenal consciousness only requires that there are at least *some* patterns --- any patterns at all --- of subjective experience *present-for-it*. It is purely about the *having* of *any* sorts of patterns of subjective experience, whether simple or complex, faint or vivid, meaningful or meaningless, fleeting or lingering (Revonsuo, 2006, p. 37).

Related to Levine’s “gap” is what Philosopher David Chalmers called the problem of the *character of conscious experience:*

Why do individual experiences have their particular nature? When I open my eyes and look around my office, why do I have *this* sort of experience? At a more basic level, why is seeing red like *this*, rather than like *that*! ….Why is the experience one way rather than the other? Why, for that matter, do we experience the reddish sensation that we do, rather than some entirely different sensation, like the sound of a trumpet? (Chalmers, 1996, p. 5).

So just like Broad wondered how the chemistry of ammonia and the functions of the brain could explain the *particular smell* of ammonia and Levine pondered the explanatory gap between the brain and the *specific qualities* of subjective experience, Chalmers wants an explanation of how brain processes could explain why pain *feels* the particular way “pain” feels. And why are there differences between the subjective feel of “red,” the note “C-sharp,” and feeling “happy” or “sad”? So all these writers are asking similar questions: how does the brain create the specific qualities of subjective experience?

**Figure 1:**
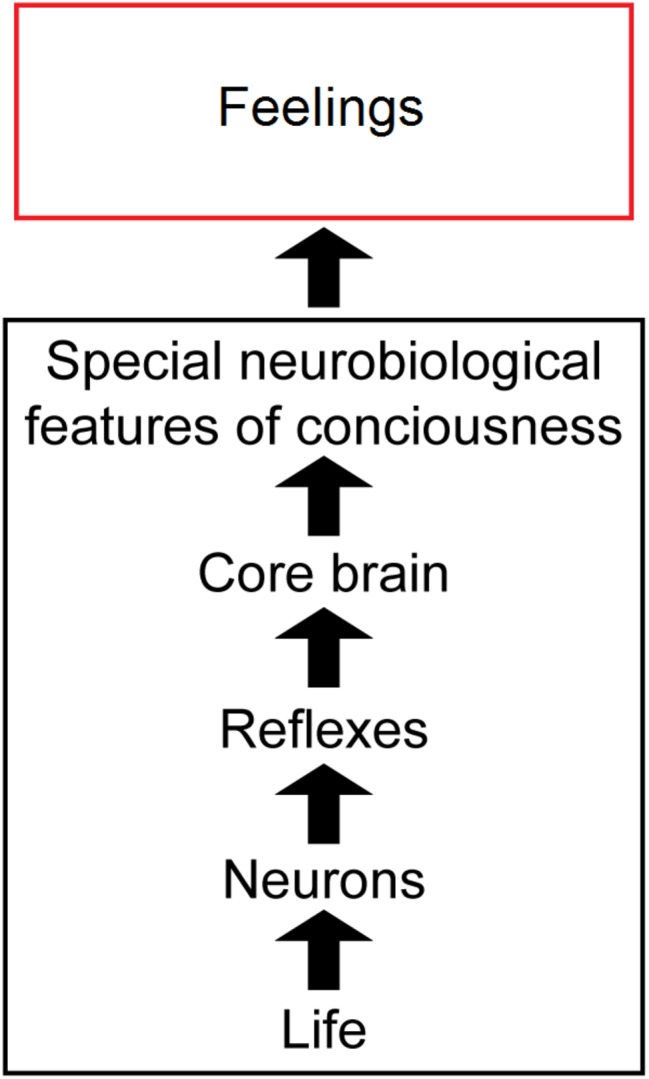
Overview of our theory of consciousness. The sequence from physical life through conscious feelings. Life and the special features of consciousness are emphasized most. The images are reproduced with the permission of the copyright holder Mount Sinai Health System.

In this paper, we argue that several biological principles are needed to explain the complex biological mechanisms behind feelings (Figure 1). First, animal feelings are built on the foundations of the general features of life, plus additional simple and complex neuronal reflexes, and then on core-brain functions (Feinberg and Mallatt, 2016a, 2018a; Lacalli, 2018). Next, to these are added a set of “special neurobiological features” of consciousness, which are diverse, distributed, and in fact are unique to conscious brains and indeed *unique in all of nature.* But the multifactorial and diverse bases of feeling, in addition to its biological uniqueness, make the connections between the neural mechanisms of feeling and the experience of feeling difficult to define in a discrete or parsimonious manner. Nonetheless, the connections can be understood in an unmysterious, scientific way. Note that we use the term “feelings” to mean *all* kinds of experiences, not just the affective or emotional experiences as it is sometimes used.

## Some Reasons Why Explaining the “Gap” is so Difficult: The Many, Diverse Mechanisms Behind Consciousness

### Mechanisms Include General Life Functions and Reflexes

The general functions of life include growth, metabolism, coded information, adaptation, and more (Mayr, 2004). That the general life functions contribute to consciousness is supported by the numerous commonalities between these functions and conscious feelings (Table 1). Namely, both life and consciousness are embodied, both are processes, both are unique features of complex, hierarchically organized systems (cellular and neural, respectively), and both are the result of their systems’ subparts and the subparts’ interactions (Atmanspacher, 2015; Nunez, 2016; Feinberg and Mallatt, 2016a; Solms, 2019). In fact, one can view consciousness as an elaboration of the interactions of multicellular life’s most complex cells, the neurons.

**Table 1:** Features that consciousness shares with life.

Cell as a key unit
Embodiment: in a body with a boundary
Process: mechanisms and functions are performed by actions of the individual parts
System: in which interactions between the parts are critical
Hierarchy: the system has different, interacting levels, of increasing complexity

Especially important for understanding the basis of feeling is that both life and feeling are *embodied*. Thus, as each living organism has a body with a boundary from the outer world, so consciousness needs a body for a subject to have a personal (first-person) point of view (see for instance, Thompson, 2007).

There are critical ways in which consciousness goes beyond basic life, in needing a body with many cells, neurons that communicate by action potentials and synapses, nervous reflexes, and even a basic, core brain. These necessary elements are *not sufficient* for consciousness, however, because some animals have them yet seem to operate only by reflexes and basic motor programs (most worms and slugs: Klein and Barron, 2016; Feinberg and Mallatt, 2016a, 2018a). Something more is necessary.

### The Mechanisms of Consciousness Require the Special Neurobiological Features

**Figure 2:**
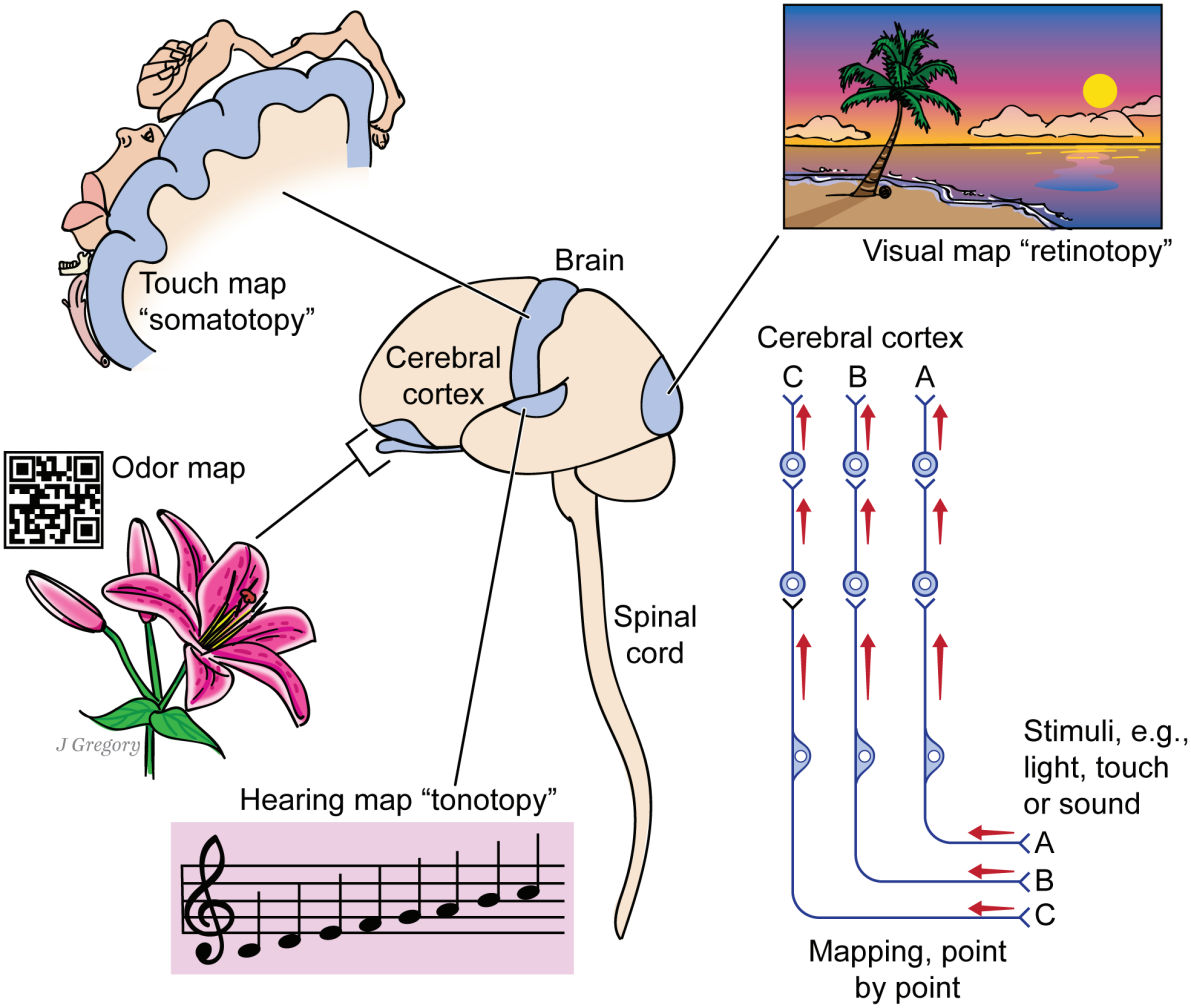
The image-based kind of consciousness involves the brain mapping the sensed world. A human brain and spinal cord are shown at center. The cerebral cortex processes mapped signals from different senses: from vision, whose mapping of the visual field is called retinotopy; from the touch senses whose mapping of the body parts is somatotopy; from the sense of smell, which forms an odor map like a bar code; and from the auditory sense, whose mapping of sounds by pitch is tonotopy. The picture at right shows that each kind of sensory signal reaches the cerebral cortex through a chain of several neurons, while maintaining its point-by-point mapping through the entire route. Some animals have no cerebral cortex yet have such maps in other higher brain centers. (From *Consciousness Demystified*, MIT, 2018. The images are reproduced with the permission of the copyright holder Mount Sinai Health System.)

This “something more” is a set of *special neurobiological features* of complex brains, which combined with the more basic life functions, reflexes, and core brain, create consciousness. We deduced these special features from simple premises (Feinberg and Mallatt, 2018a,b). First, we assumed all animals with brains that organize their sensory inputs into detailed, multisensory, and mapped representations of the world and body can consciously experience sensory mental images (Figure 2). Indeed such neural maps—more multisensory, unified, and complex than known computers and artificial intelligence can achieve (LeCun et al., 2015)—are often considered to be a marker for primary consciousness (Edelman, 1992; Damasio, 2010). Second, we assumed that all animals capable of global operant learning—learning new, complex survival behaviors from experience based on rewards and punishments—can feel positive and negative effects, and thus, they have the affective type of consciousness (akin to felt human emotions). Our logic for this was that if an animal first reacts to the rewarding or punishing stimuli, then shows through complex behavior that it remembered the stimuli, it must have felt them in the first place (see Feinberg and Mallatt, 2016a; and Bronfman et al., 2016 added important considerations). Third, we identified the animal clades that fit these criteria—all the vertebrates, cephalopod molluscs, and arthropods—and examined their neurobiology to uncover still more neural and behavioral features associated with consciousness. Through these three steps, we built our set of special features of consciousness (Table 2).

**Table 2:** The special neurobiological features of consciousness (mostly after Feinberg and Mallatt, 2018a).

Neural complexity (more than in a simple, core brain) Brain with many neurons (>100,000?) Many subtypes of neurons
Elaborated sensory organs Eyes, receptors for touch, taste, smell
Neural hierarchies with neuron-neuron interactions Extensive reciprocal communication in and between pathways for the different senses Brain’s neural computing modules and networks are distributed but integrated, leading to local functional isolation plus global coherence (Nunez, 2016; Mogensen and Overgaard, 2017) Synchronized communication by brain-wave oscillations The higher levels allow the complex processing and unity of consciousness Hierarchies that let consciousness predict events a fraction of a second in advance
Pathways that create mapped mental images or affective states Neurons are arranged in topographic maps of the outside world and body structures Valence coding of good and bad, for affective states Feed into pre-motor brain regions to motivate, choose, and guide movements in space
Brain mechanisms for selective attention and arousal
Memory, short-term or longer

Invertebrate consciousness is debated, so we will consider it closer here. Actually, consciousness in the cephalopod octopuses and squids is not so controversial because investigators recognize the sharp senses of these animals, the close attention they pay to their surroundings, and their advanced learning abilities (Mather and Carere, 2016; Godfrey-Smith, 2016a). The real controversy is about whether insects and other arthropods have consciousness. Although we exhaustively assembled the evidence for this in our previous works (Feinberg and Mallatt, 2016a, 2018a; also see Klein and Barron, 2016), we will review that evidence we found most suggestive. Bees can see, learn, and remember a complexly patterned target for finding food (suggesting image-based consciousness: Fauria et al., 2000), and they pass the “judgment bias test” (i.e., are more likely to choose an ambiguous cue toward a reward if they just received a sample of that reward: Perry et al., 2016). Judgment bias is a standard test for positive affective consciousness in animals, and passing it is said to indicate that one feels an anticipated reward. Lest it seem that arthropod consciousness only applies to the relatively large-brained bees, jumping spiders also show evidence for it. These spiders can follow the route to their prey after just temporarily seeing that prey from a distance, suggesting they form mapped, mental images (Jackson and Cross, 2011; Perry and Chittka, 2019). Also, all arthropod brains are built on the same plan and have the same parts (Strausfeld, 2012), even that of the earliest fossil arthropods from 520 million years ago (Ma et al., 2012). This suggests all arthropods have had consciousness from the beginning.

Let us cover the special features in Table 2 more systematically. We have documented how this set of vertebrate-arthropod-cephalopod features is absent from animals with simpler nervous systems: namely from all the other invertebrates, the nonconscious worms, jellyfish, clams, sea squirts, sea stars, etc. (Feinberg and Mallatt, 2013, 2016a). The features include an explosion of special senses (image-forming eyes, acute hearing, and keen smell) and of neuron types; many new neural processing subsystems; more integration of information from the different senses; more hierarchic levels of neurons for processing information; extreme reciprocal and oscillatory cross-communication between the lower and higher levels and between participating brain regions (Lamme, 2006; Koch et al., 2016; Northoff, 2016; Nunez, 2016; Grossberg, 2017); more effective attention; and more memory. From these features arise the extraordinary neurobiological system-properties of complex brains in a way comparable to how life naturally arises from the interactions of its subcellular and cellular components. But these neurobiological features are even more remarkable for the creation of consciousness than are those that create life: together they are unique to conscious brains – and indeed are *unique in all of* nature – so it should come as no surprise, nor present an unfathomable mystery, that they produce something unique in nature like feelings.

This leads to the question of the adaptive role of consciousness in animals that have it. We analyzed the literature and came up with a list of adaptive functions (Table 3; Feinberg and Mallatt, 2018a). Foremost among these are that consciousness efficiently codes and organizes large amounts of sensory input into a unified set of phenomenal properties for choosing among many active responses, and that its unified simulation of the subject’s body moving through complex environments allows survival and reproduction in the wild (Cabanac, 1996; Seth, 2009; Klein and Barron, 2016; Merker, 2016).

**Table 3:** Some adaptive roles of consciousness (mostly after Feinberg and Mallatt, 2018a).

Organizes large amounts of sensory input into a set of phenomenal properties for action choice
Its unified simulation of the sensed world directs behavior in this world
It ranks sensed stimuli by importance, by assigning affects to them, making decisions easier
Allows flexible behavior because it sets up many different behavioral choices
Allows easily adjustable behavior because it predicts the consequences of one’s actions into the immediate future (Perry and Chittka, 2019; Solms, 2019)
Deals well with new situations, to meet the changing challenges of complex environments

**Figure 3:**
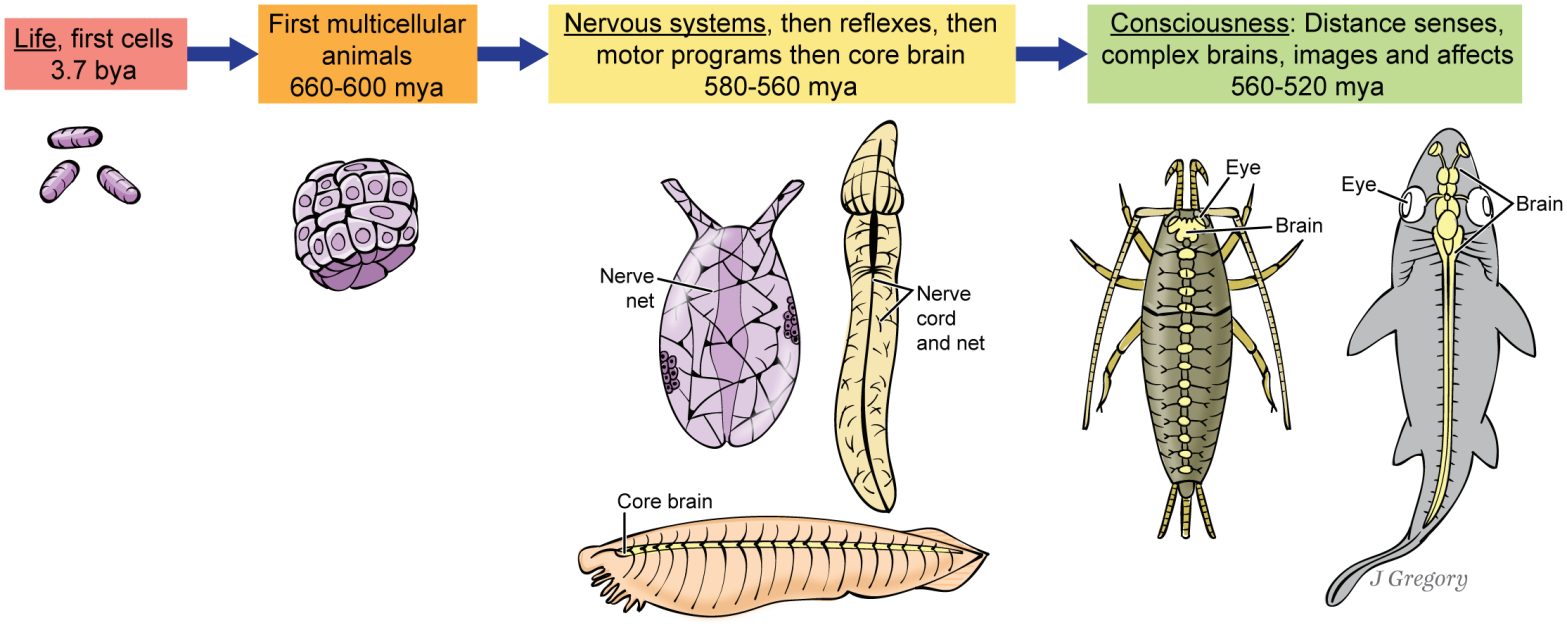
Proposed stages in the evolution of consciousness, as an uninterrupted sequence. The three animals at the ‘Nervous system’ stage at center are hypothetical, but based roughly on a sea anemone, a hemichordate acorn worm, and the fish-like invertebrate, amphioxus. The two animals at far right are a bristletail insect and a shark. (From *Consciousness Demystified*, MIT, 2018. The images are reproduced with the permission of the copyright holder Mount Sinai Health System.)

We claim that consciousness evolved naturally, so this demands an account of *when* consciousness appeared, without any inexplicable gaps in the evolutionary sequence. Indeed, in the fossil record, we traced the evolution of consciousness and brains in an unbroken chain from blind and brainless marine worms to the first arthropods and vertebrate fish in the Cambrian seas, from about 560 or 540–520 million years ago (Feinberg and Mallatt, 2016a,b, 2018a,b). This sequence is shown in Figure 3. The stimulus would have been the rise of Earth’s first animal-on-animal predators. That led to natural selection for sharp vision and other distance senses that mapped space into a detailed, panoramic mental image to deal with the predation. The images also allowed animals to navigate accurately through complex environments. Furthermore, the predation led to selection for the affective “emotions” to drive elaborate escapes, attacks, approach behaviors, etc. (Merker, 2005; Plotnick et al., 2010; Trestman, 2013; Klein and Barron, 2016; Godfrey-Smith, 2016b).

So far, here are our conclusions about the mechanisms for consciousness. We deduced first that feelings uniquely connect to the embodied *life* of the organism, and second, they have a unique neurobiology through the set of *special features*. By contrast, most other scientific explanations of consciousness include the special features alone, which are often called the neural correlates of consciousness (e.g., Seth et al., 2005; Koch et al., 2016). However, we feel it necessary also to include the foundations of consciousness in life functions, which require only normal physics, chemistry, and evolutionary processes, to remove a big part of the “mystery” from consciousness and from its mechanisms.

### The Mechanisms of Consciousness Are Diverse

Many different theories address the causes and mechanisms of consciousness. Some promote single-factor causes: e.g., panpsychism^1^; consciousness as a fundamental force (Chalmers, 1996); and quantum microtubules (Hameroff and Penrose, 2014). Other theories recognize consciousness to be complex and multifactorial but emphasize a major factor or approach: e.g., a global workspace (Baars et al., 2013); information (Tononi and Koch, 2015; Nunez, 2016); cognitive and computational aspects (Dehaene, 2014; Piccinini, 2015; Dehaene et al., 2017); reciprocal and oscillatory neuronal communications (Lamme, 2006; Koch et al., 2016); the attentional aspects (Tsuchiya and van Boxtel, 2013; Graziano and Webb, 2015); instinct (Gazzaniga, 2018); complex new physics (Primas, 2007; Nunez, 2016; Torday and Miller, 2018); predictive properties (Solms, 2019); and contextual emergence in systems theory (Atmanspacher and beim Graben, 2009; Atmanspacher, 2015). We call these the “major-mechanism theories.” While these theories all make valid contributions to explaining the mechanisms of consciousness, our findings indicate that consciousness is even more diverse than any one of them proposes. Here, we summarize the remarkable diversity of consciousness, from multiple perspectives (Table 4).

**Table 4:** Ways in which consciousness is diverse.

A. Three subtypes Exteroceptive Affective Interoceptive
B. Brain regions (mammal example) Cerebral cortex (mapped images) Subcortical (affects)
C. The coding varies: mapped representations of space versus valence-coding
D. Hubs and nodes are widely distributed within brains
E. In different animal groups Vertebrates Arthropods Cephalopods

To begin with, we found that sensory consciousness, from the standpoint of its neural and functional properties, should be divided into three partially overlapping domains or subtypes: exteroceptive, affective, and interoceptive. Exteroceptive phenomenal properties are created from mapped, sensory mental images of the world (from what is seen, heard, smelled, etc.); affective feelings are internal, valenced feelings, both positive and negative (as in emotions); and interoceptive phenomenal properties are in-between, including both mapped sensory representations of the body’s organs and the affective feelings that protect somatic functions.

Most existing theories tend to cover just one of these subtypes, such as the exteroception-based theories of Edelman (1989, 1992) and Tsuchiya et al. (2015); emotion-centered theories of Denton (2006); Damasio (2010); Nummenmaa et al. (2018), and Solms (2019); and interoception-based studies of Craig (2010) and Vierck et al. (2013). We, on the other hand, cover all three subtypes, considering both their differences and commonalities.

Not only are these three domains different manifestations of consciousness but they also, beyond sharing the special neurobiological features, have *different neural architectures*. In mammals, for example, exteroceptive phenomenal properties stem more from the cerebral cortex, whereas affective feelings stem more from subcortical parts of the cerebrum, the hypothalamus, and brain stem. This two-site interpretation is not fully accepted, as some investigators say all types of mammalian consciousness stem from the cerebral cortex (e.g., Craig, 2010; LeDoux and Brown, 2017; Nummenmaa et al., 2018). However, it is supported by the fact that children born with little or no cerebral cortex (a condition called hydranencephaly) show strong emotional behaviors, as do mammals from which the cerebral cortex was experimentally removed (Panksepp, 2005; Aleman and Merker, 2014). For more discussion, see Feinberg and Mallatt (2018a).

Exteroceptive circuits are organized to encode a mapped representation of space (Edelman, 1989; Stein and Meredith, 1993), whereas affective circuits encode positive and negative valences instead (Shao et al., 2017; Adolphs and Anderson, 2018; Corder et al., 2019).

As more evidence that consciousness is not monolithic, its neural substrates are *widespread* within brains. This is now widely recognized, and Nunez (2016) gives an especially good account. For us, it is seen best for the affective circuits in vertebrates, which involve interconnected hubs spread over such distant areas as the amygdala and basal forebrain, midbrain, hypothalamus, and habenula of the diencephalon (Hu, 2016). And the reticular formation, which distributes through much of the vertebrate brain stem, projects very widely to control the attention and arousal aspects of consciousness (Schiff, 2008; Brodal, 2016). In insects, which we likewise deduced to have consciousness, the neurons for arousal and attention also distribute widely through the brain (Van Swinderen and Andretic, 2011).

**Figure 4:**
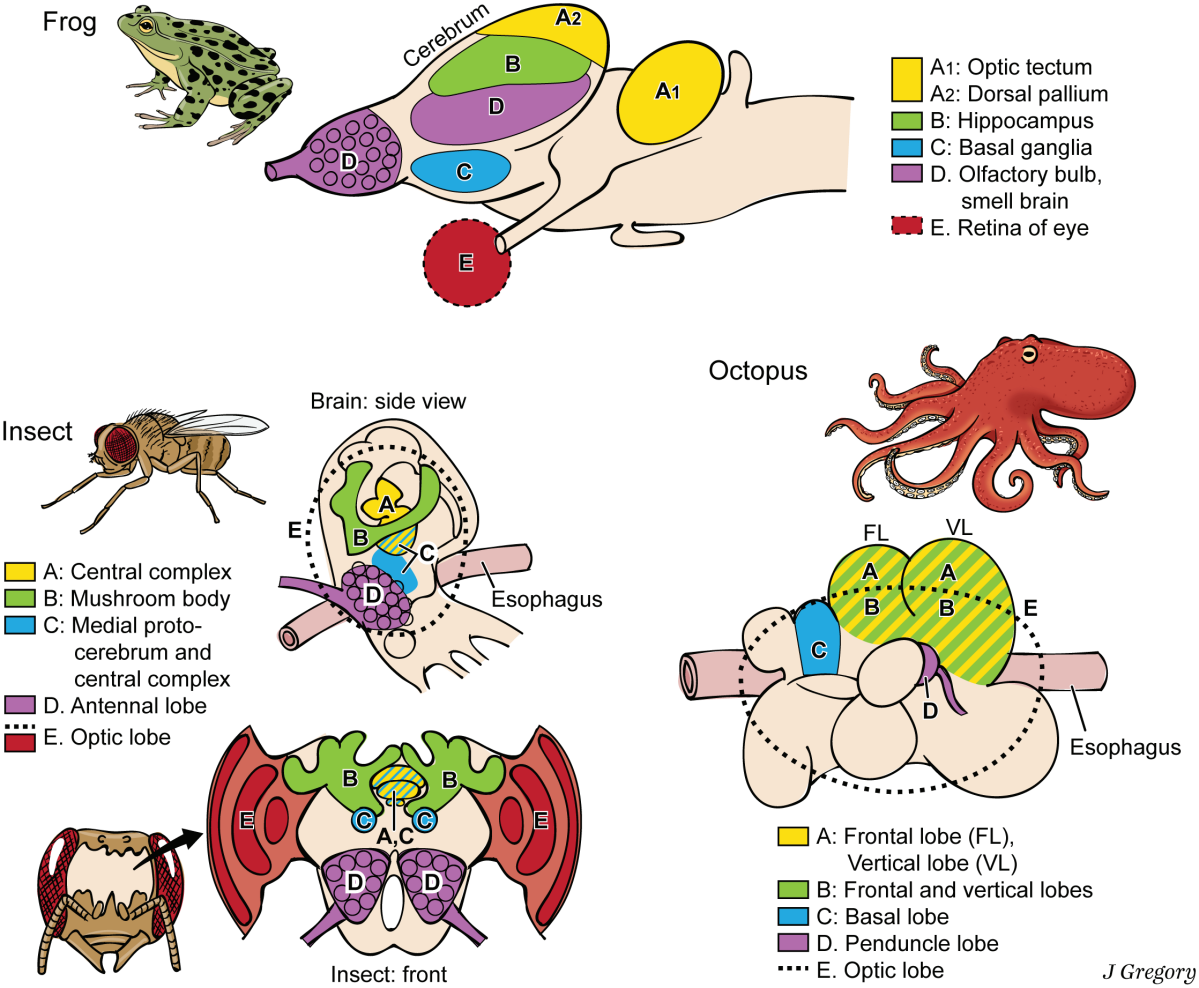
Dissimilar brains of three different taxa of animals with consciousness. The areas with similar functions are colored the same in the different brains. The general code is: A, image-based consciousness; B, memory; C, pre-motor center; D, smell processing; and E, visual processing. (From *Consciousness Demystified*, MIT, 2018. The images are reproduced with the permission of the copyright holder Mount Sinai Health System.)

Still more demonstration that phenomenal properties and their neural substrates are extremely diverse and widespread comes from cross-species comparisons. The vertebrates, arthropods, and cephalopods evolved brains entirely independently, from a brainless common ancestor (Northcutt, 2012). Thus, they all evolved the brain regions for consciousness separately, by convergent evolution (Edelman, 2016). For a vertebrate, an insect, and an octopus, we mapped the brain regions that associate with various aspects of consciousness, from image-based consciousness to the participating memories (Figure 4), and found that these regions look different and have different relative locations in the three animal brains (Feinberg and Mallatt, 2018a). Apparently, primary consciousness can stem from very different brain substrates.

To summarize, the multifactorial basis of conscious experience, including its foundations in life processes, its widespread neural organization, its diversity both within and across species, and its uniqueness in biology and indeed all of nature, make it exceedingly difficult if not impossible to encapsulate or “pinpoint” its biological and neurobiological substrate and cause in the way, for instance, that photosynthesis can account for energy creation in plants or that DNA can account for the mechanisms of hereditary.

But while this complexity makes it difficult to learn how brains create experience, this does not mean the process is mysterious or unexplainable. Many complex biological functions—including life itself—are multi-determined aggregate functions that cannot be reduced to a few factors, yet are nonetheless scientifically explainable (Von Bertalanffy, 1952; Mayr, 2004; Drack, 2015).

## Can the Personal (Subjective) Nature of Sensory Consciousness Have a Natural (Biological) Explanation?

Despite the phenomenal and neurobiological diversity, we propose that the subjective, phenomenal, aspects of experience can be explained using the principles of normal physics, chemistry, and biology. The question is how?

Our answer is best broken down into two parts. First, as we argued above, because phenomenal properties are built upon life, and both life and consciousness are system features of *embodied* organisms, then it follows that consciousness is personal and unique to the living organism. Seen in this light, these feelings are aggregate functions of certain complex, personal (living) brains, from which they cannot be dissociated. This is true whether we are talking about such different feelings as red, pain, hunger, or happiness. Essential is the fact that the feeling of red is a *personally specific and neurobiologically unique* feature of an individual’s particular brain state, as are all phenomenal properties. Therefore, certain complex neural features of this system, could, unmysteriously, result in a unique, personal system-feature like subjective experience, just like “life” is a personal system feature of complex living organisms. Thus, life itself provides the initial conditions for the subjectivity of experience.

But second, to this we must add the features of increased complexity and neurobiological uniqueness of the special features that are ultimately responsible for both the creation of - and the specific qualities of - subjective experience. The special features are numerous and enormously complex, but we derived them scientifically and they are entirely biologically natural (see Table 2 and the associated text). Add to this the aforementioned personal aspect of subjective experience, due to its basis in the embodied life processes of the individual organism, and then the combination of these factors—complex neurobiological uniqueness walled off within personal embodiment—makes consciousness appear “mysterious” and inexplicable by known physical law. But we did not need to invoke a “mysterious” explanation to account for the personal and unique aspects of subjectivity.

Now, what of Chalmers question about the specific character of conscious experience? Why is a color experienced differently from a sound or pain? The answer lies in the *diverse neurobiology* behind these varied subjective experiences. For instance, it is clear that the neural pathways of color processing, sound processing, pain processing, affect and so on show enormous neurobiological differences (Brodal, 2016; see Feinberg and Mallatt, 2016a, for further discussion). Electromagnetic waves of light have many different physical properties than the mechanical forces of touch, and both differ from chemical odorants, so translating all three kinds of stimuli into similar feelings would miss the special properties that make each sense so especially informative. Therefore, these diverse sensations should not – and indeed *could not* – all have the same subjective “feel.” It should come as no surprise that the phenomenal experiences created by these varied neural architectures differ in how they are subjectively experienced. In other words, the *qualitative features of phenomenal properties lie in the neural states themselves*; they are not an “additional feature” to the neural states that create them.

## Discussion

We have built the case that there are no scientific “gaps” in the neurobiology and evolution of consciousness, but rather a seamless series of transitions between levels of increasing neural differentiation, complexity, and hierarchy that lead to phenomenal consciousness. We find that with this approach we do not need to posit any new, singular or fundamental “physical” factor that explains the *unique* and *personal* nature of phenomenal consciousness. Here, we summarize the major points in this argument.

### Consciousness Has Its Foundations in Life Itself

The neurobiological mechanisms of consciousness that we identified have their foundations in embodied life and simpler nervous elements (Table 1). The later-evolving and more-complex special neurobiological features of advanced brains are built upon and derived from the general features of life, which they retain.

The role that the features of life play in the creation of consciousness has actually been noted by some philosophers who are interested in explaining consciousness (Churchland, 1996, 2013; Thompson, 2007). For instance, Evan Thompson argued that the failure to appreciate the relationship between life processes and consciousness mistakenly draws an “unbridgeable divide” between the physical brain on the one hand and experience (feeling) on the other, a mistake that in essence disregards all the general biological features that can help explain consciousness and how subjectivity is created:

I have argued that the standard formulation of the hard problem is embedded in the Cartesian framework of the “mental” versus the “physical,” and that this framework should be given up in favor of an approach centered on the notion of life or living being. Although the explanatory gap does not go away when we adopt this approach, it does take on a different character. The guiding issue is no longer the contrived one of whether a subjectivist concept of consciousness can be derived from an objectivist concept of the body. Rather, the guiding issue is to understand the emergence of living subjectivity from living being, where living being is understood as already possessed of an interiority that escapes the objectivist picture of nature (Thompson, 2007, p. 236).

Thus, embodied life ultimately gives subjectivity its personal nature. But as Thompson notes, the explanatory gap by no means goes away simply because consciousness is a feature of life. Life partly fills that gap but something more is needed - something uniquely neurobiological is required to explain the transition from life to the unique and personal features of phenomenal consciousness. That ‘added something’ would be the special neurobiological features.

### The Special Features Are Critical to the Creation of Consciousness

Like life, consciousness is an aggregate property of certain less-complex properties. But there is a difference. Cells, tissues, or organs can still be “alive” individually because they do not rely on higher hierarchical levels for their “living properties.” For consciousness, by contrast, while its lower-level neural elements like individual neurons or reflex arcs are all independently alive, the aggregate property of “consciousness,” which includes *both* living properties and complex, hierarchical, neural properties, does not emerge until much higher levels are added (the special features of Table 2).

### The Neurobiology of Consciousness Is Diverse

According to our approach, many different neurobiological architectures are capable of creating different manifestations of phenomenal consciousness. Although these share the special features of Table 2, their great diversity both within brains and across animal phyla and their multifactorial and neurobiologically widespread origins in brains *make it impossible to discretely, exactly, and simply define consciousness’s biological and neurobiological basis*. Thus, we cannot explain *all* feeling states as derived from the same neurobiological elements. Even if it were “one thing,” the aggregate phenomenon of consciousness has many diverse aspects and pathways. And many different major-mechanism theories may apply (global workspace, informational, computational, etc.). The recognition that consciousness is even more diverse and widespread than previously realized can encourage the many existing theories to come together and integrate their approaches.

### Consciousness Is Neurobiologically Unique

The set of special features is novel and unique in biology, and hence, they contribute to the uniqueness of consciousness in nature. Consciousness is best viewed as *a unique but multi-determined aggregate system feature of life and complex brains.* This uniqueness makes consciousness difficult to study because consciousness cannot be compared to anything else, but it is not mysterious. And this view does not *require* that we explain all the mechanisms of subjective experience within the solution. The “gap” is best explained by the *combination* of several critical factors – the personal embodiment of subjectivity that derives from life, with the unique, complex, and diverse neurobiological features that contribute to consciousness.

Table 5 summarizes, in simplest form, how we fill the explanatory gap and demystify consciousness.

**Table 5:** Consciousness is demystified, as.

1. Personal Inherited from embodied life
2. Unique Distinctive combination of many biological and neural features (Tables 2, 3)
3. Diverse Too neuroanatomically distributed and architectonically varied within brains and across species (Table 4) to be explained by a single cause or overall mechanism

## Author Contributions

For this paper, TF focused more on the theory, philosophy, and neurobiology and JM focused more on the neurobiological and evolutionary aspects.

### Conflict of Interest Statement

The authors declare that the research was conducted in the absence of any commercial or financial relationships that could be construed as a potential conflict of interest.

## Footnotes

^1^https://plato.stanford.edu/entries/panpsychism/
